# Evaluation of Wound Healing Effect of Topical Phenytoin on Excisional Wound in Albino Rats

**DOI:** 10.4103/0975-1483.62215

**Published:** 2010

**Authors:** AA Hasamnis, BK Mohanty, Muralikrishna, S Patil

**Affiliations:** *Department of Pharmacology, GSL Medical College, Rajahmundry, India*

**Keywords:** Phenytoin cold cream, area of wound healing, percentage healing of wound area, complete epithelization

## Abstract

**Objectives:**

Wound healing is a significant healthcare problem in today’s medical practice. Despite extensive treatment modalities that are supposed to hasten the wound healing process, the outcomes of existing methods are far from optimal. One such agent that has been tried previously and found controversial in wound healing is phenytoin. In this study, the wound-healing efficacy of phenytoin was investigated in albino rats.

**Materials and Methods:**

20 male Wistar albino rats were subjected to excisional wounds measuring 500 mm^2^ on the back and then randomized to two groups (n = 10): Control group (A) and treatment group (B). The control group received no drug treatment till the end of the study. 1% Phenytoin cream was applied to the wounds of rats in the group B and continued till the 16^th^ day of the study. The areas of wounds were measured on the Days 4, 8, 12, and 16 of the experiment. The percentages of the healing wounds were calculated by Walker formula after measurement of the wound area. The total number of days required for complete epithelization of wounds was noted in each group.

**Results:**

Statistically significant reduction (*P* < 0.05) in average wound area was seen in Group B (*P* value=0.0017, 0.0001, 0.0001, 0.0001), respectively, on Days 4, 8, 12, and 16 of the experiment in comparison to Group A. The average number of days required for complete epithelization of wound area was less in Group B as compared to Group A (*P*=0.0120). The difference was statically significant

**Conclusion:**

In the present study, topical phenytoin accelerated healing of excisional wound in albino rats.

## INTRODUCTION

Non-healing and chronic wounds are a significant healthcare problem in today’s medical practice.[1] Wound healing is the process of restoration of physical integrity of internal or external body structures and involves complex interactions between the cells and various other factors.[2] The healing process consists of a sequence of overlapping events including inflammatory responses, regeneration of the epidermis, shrinkage of the wound and finally connective tissue formation, and remodeling.[2] Appropriate treatment and wound care accelerate the healing process and prevent infection and chronicity of the wound.[3] Different methods and approaches have been used to achieve shorter wound healing times.[1] Despite extensive efforts to improve wound healing, the outcomes of existing methods are far from optimal.[2]

One such agent that has been tried in wound healing is phenytoin. Phenytoin (diphenylhydantoin) was introduced into therapy in 1937 for the effective control of convulsive disorders.[4] A common side effect with phenytoin is the development of fibrous overgrowth of gingiva.[5] This apparent stimulatory effect of phenytoin on connective tissue suggested an exciting possibility for its use in wound healing.[3]

Previous clinical studies have shown topical phenytoin to promote healing of ulcers but the efficacy of phenytoin in treatment of such conditions is still controversial and many of the existing clinical studies have methodological flaws, such as inappropriate statistical analysis, inadequate control groups, and the absence of randomization and double-blinding.[6] The present literature available indicates that topical phenytoin deserves further investigation as a wound healing agent in preclinical studies in a controlled environment.[3] Hence, this study was conducted to evaluate the effect of phenytoin on the wound healing process on laboratory rats.

## MATERIALS AND METHODS

The experiment was conducted on 20 male wistar rats weighing 200-300 g. The animals were caged individually in a controlled environment (temperature 30°C) with a 12 h artificial light cycle. Food and water were available ad libitum to the rats. For carrying out the experiment, Institutional Animal Ethics Committee permission was taken.

Prior to creating excisional wounds the rats were anesthetized by intraperitoneal injection of ketamine (50 mg/kg).[2] Then the animals were shaved on the back and the skin was disinfected using cotton and alcohol wipes. Using sterile surgical instruments round full thickness skin wounds measuring 500 mm^2^ were created in the paravertebral area, 1.5 mm from midline on the back of rats, and then thoroughly disinfected using povidone iodine.[2,3]

All the 20 rats were then randomly allocated to two groups, each group containing ten rats each, namely control (Group A) and phenytoin treatment (Group B). For the preparation of 1% phenytoin cream, 1 g of phenytoin powder was added to 99 g of cold cream and it was then applied over the wound surface of Group B rats twice daily.[7] Control Group A received no drug treatment. The applications of phenytoin continued for 16 days from the start of the experiment.

To measure the contracture of the wound, a transparent plastic paper was placed on the location of wound and its shape was drawn on the same paper with a marker and then matched with the graph paper for finding the area of the wound (expressed in mm^2^).[2,8]

Wound healing was measured on Days 4, 8, 12 and 16 of the experiment.[3] Percentage of wound healing was calculated according to the Walker formula.[7]

Percentage of wound area = Wound area on day X/Wound area on day 1 × 100

Percentage of wound healing = 100−Percentage of wound area

The total number of days required for complete epithelization of the wound was noted in each rat in both the groups (e.g. fall of scab without any raw area).[2,8]

### Statistical analysis

Data were analyzed using SPSS version 13.All the results were expressed as Mean ± S.D. “Unpaired *t*-test” was used to compare area of wound healing in both the groups (A and B) on Days 4, 8, 12, and 16 of the experiment. “Unpaired *t*-test” was used to compare time required for complete epithelization of the wound in between the groups. *P* < 0.05 was considered to be significant.

## RESULTS

### Average area of the wound measured in sq mm expressed as Mean ± S.D

The observations of the study showed that when average wound area was compared between Group A (474 ± 12.07, 379 ± 21.02, 293 ± 18.43, 194.80 ± 11.62) and Group B (386 ± 69.31, 300 ± 11.51, 221.40 ± 15.23, 113.20 ± 12.24) on Days 4, 8, 12 and 16, respectively, the results were statistically significant (*P* < 0.05) [Table 1].

**Table 1:** Average wound measurements in the groups

Groups (n = 10)	Day 4	Day 8	Day 12	Day 16
Group A	474 ±	379 ±	293 ±	194.80 ±
Wound area (mm^2^)	12.07	21.02	18.43	11.62
Group B	386 ±	300 ±	221.40 ±	113.20 ±
Wound area (mm^2^)	69.31	11.51	15.23	12.24
*t* value	3.7669	10.4396	9.4722	15.2904
*P* value	<0.0017*	<0.0001*	<0.0001*	<0.0001*

When the average area of the wound measured in sq mm expressed as mean ± S.D was compared between the group A and group B, it was highly statistically significant on Day 4, Day 8, Day 12 and Day 16 of the experiment.

(*Statistically significant)

### Days required for complete epithelization

The observation of the study also revealed that the average number of days required for complete epithelization of wound in Group A rats (23.00 ± 2.26) was significantly higher than the period required for complete epithelization in Group B rats (20.60 ± 1.51) and the difference was statistically significant (*P* < 0.05) [Table 2].

**Table 2:** Average time taken for epithelization in the groups

Groups (n = 10)	Days required for epithelization
Control group A	23.00 ± 2.26
Treatment group B (phenytoin)	20.60 ± 1.51
*t* value	2.7941
*P* value	<0.0120*

The average number of days required by the rats in both the groups for complete epithelization of the wound is shown. The values are expressed as mean ± S.D.

The *P value is highly significant when comparison was done between groups A and B for days required for complete epithelization. (*Statistically significant)

## DISCUSSION

In 1939, Kimball first observed that gingival hyperplasia occurred in some patients treated with phenytoin. This stimulated the study regarding the potential use of phenytoin in wound healing.[9] Shapiro carried out the first clinical trial in 1958 and found out that periodontal patients with surgical wounds who were pretreated with oral phenytoin had less inflammation, less pain, and accelerated healing as compared to controls.[10] Subsequently, it was seen that phenytoin promoted the healing of dental extraction sockets and also increased the tensile strength of skin wounds.[11,12] There are studies and case reports about the use of topical phenytoin for a wide variety of soft tissue infections and ulcers.[6]

Menezes *et al*., Bogaert *et al*., and Bansal and Mukul showed the usefulness of topical phenytoin in lepromatous trophic ulcers.[13–15] El Zayat examined the effects of topical phenytoin on decubitus ulcers and missile wound injuries and found it to be useful.[16] Phenytoin showed positive results in the treatment of gluteal abscesses secondary to intramuscular injection in a study conducted by Lodha *et al*.[17] Pendse *et al*. found antibacterial activity of phenytoin in the wound healing process.[18]

The mechanism by which phenytoin accelerates the wound healing process is unknown. Clinical studies suggest that stimulation of fibroblastic proliferation, enhancing the formation of granulation tissue, decreasing collagenase activity (by reducing its production or secretion or both), promoting deposition of collagen and other connective tissue components, decreasing bacterial contamination, and by decreasing the formation of wound exudate to be some of the ways by which topical phenytoin hastens the healing process.[3,19,20] Biopsies of phenytoin-treated open wounds show neovascularization and collagenization.[19] Local pain relief due to membrane-stabilizing action of topical phenytoin therapy has been observed in several studies.[20,21]

Topical application of phenytoin results in direct access of the drug to the target site and avoids the risk of getting systemic side effects.[21] The findings of the above-mentioned study were taken into consideration for selecting the route of administration of phenytoin for wound healing in the present study.

Even with all the positive data available, the role of topical phenytoin in the wound healing process is still controversial and efficacy is doubted due to the lack of multicentric, double blind nature of the earlier studies. The use of topical phenytoin for wound healing is not approved by the USA Food and Drug Administration (FDA) at present.[3] In a paper published by FDA on orphan products development (1984), phenytoin was listed as an important agent for wound healing, subject to confirmation by fresh preclinical, and multicentric double blind clinical studies.[4]

In the present study, it is seen that phenytoin hastens the healing of excisional wound in albino rats.These findings support the findings of earlier studies.

## CONCLUSION

In the present study, topical phenytoin accelerated healing of excisional wound in albino rats.
